# Natural clearance time and effect of manuka honey body wash and oral probiotics on colonization of *Candidozyma auris* (*Candida auris*) among residents of older adults homes in Hong Kong

**DOI:** 10.3389/fpubh.2026.1899016

**Published:** 2026-08-05

**Authors:** Edmond Siu-Keung Ma, Raymond Wai-Man Lai, Vivien Wai-Man Chuang, Bianca Suet-Ying Shing, Leo Lui, Enoch Hsu, Emily Kiu, Hong Chen, Edwin Lok-Kin Tsui

**Affiliations:** 1 Department of Health, Infection Control Branch, Centre for Health Protection, Hong Kong, Hong Kong SAR, China; 2 Hospital Authority, Hong Kong, Hong Kong SAR, China; 3 Department of Health, Centre for Health Protection, Hong Kong, Hong Kong SAR, China

**Keywords:** antimicrobial resistance, *Candida auris*, decolonisation, epidemiology, infection control

## Abstract

**Background:**

*Candidozyma auris* (*C. auris*) has emerged globally as a multidrug-resistant healthcare-associated fungal pathogen and is most commonly associated with bloodstream infections, which have high global mortality rates. However, little is known about the natural clearance time and effectiveness of decolonization therapy. We reported the natural clearance duration of *C. auris* and assessed whether decolonization using manuka honey baths and oral probiotics containing *Lactobacillus rhamnosus GG* and *Saccharomyces boulardii* can reduce colonization time.

**Methods:**

We reviewed the demographic characteristics and outcomes of *C. auris* cases staying in RCHE in Hong Kong from 1 June 2019, when they were cleared of the fungus or died during the follow-up period up to 31 December 2024. Clearance of *C. auris* was defined as culture negative for at least three consecutive specimens collected monthly at the RCHE. We compared the clearance time of *C. auris* with and without decolonization using Kaplan-Meier methods.

**Results:**

A total of 215 cases and 190 controls were analyzed with no major differences in demographic characteristics. The Kaplan-Meier analysis estimated that the median time until 50% of RCHE residents cleared *C. auris* colonization was 224 days for the intervention group and 427 days for the control group. The cases and controls showed no statistical difference in *C. auris* clearance duration.

**Conclusion:**

The long natural clearance time for *C. auris* poses pressure on infection control practices in RCHE. Further research in randomized controlled trials and other treatment modalities is encouraged to reduce its transmission.

## Introduction

*Candidozyma auris* (*C. auris*) has emerged globally as a multidrug-resistant healthcare-associated fungal pathogen since its first identification in Japan in 2009. Unlike *Candida albicans,* which mainly colonizes the gastrointestinal and genitourinary tracts of healthy individuals, *C. auris* is thought to predominantly colonize the skin but can also be isolated from the gut, oral, and esophageal mucosa of infected individuals (1). Clinical *C. auris* isolates have been identified from various specimen types including sterile body fluids, respiratory secretions, urine, bile, tissues, wounds, and mucocutaneous swabs (2). It is commonly associated with bloodstream infections with high global mortality rates ranging from 30 to 60% (3). High patient mortality and its intrinsic resistance to one or more classes of antifungal drugs available in the clinic have posed a significant disease burden (4, 5). The World Health Organization has listed *C. auris* in the critical group of fungal priority pathogens to guide research, development, and public health action (6). RCHE are more prone to acquire multi-drug-resistant organisms due to the presence of chronic illness, being immunocompromised or dependent, and frequent hospitalization (7, 8). In a local prevalence study of 26 RCHEs with 1,529 residents in Hong Kong, the prevalence rates of methicillin-resistant *Staphylococcus aureus* and Carbapenem-resistant *Acinetobacter* were 33.9 and 8.1%, respectively (9). Similarly, risk factors for *C. auris* infections include older adults, diabetes mellitus, recent surgery, the presence of an indwelling medical device, an immunosuppressed state, chronic renal disease, hemodialysis, and broad-spectrum antifungal drugs (3). Therefore, outbreaks have occurred in healthcare facilities, particularly in long-term care hospitals and nursing homes and in the community (10–13). Given the propensity of *C. auris* to cause outbreaks, halting transmission primarily depends on good hand hygiene compliance, coupled with standard and contact precautions, and thorough cleaning and disinfection of the environment.

**Figure 1:**
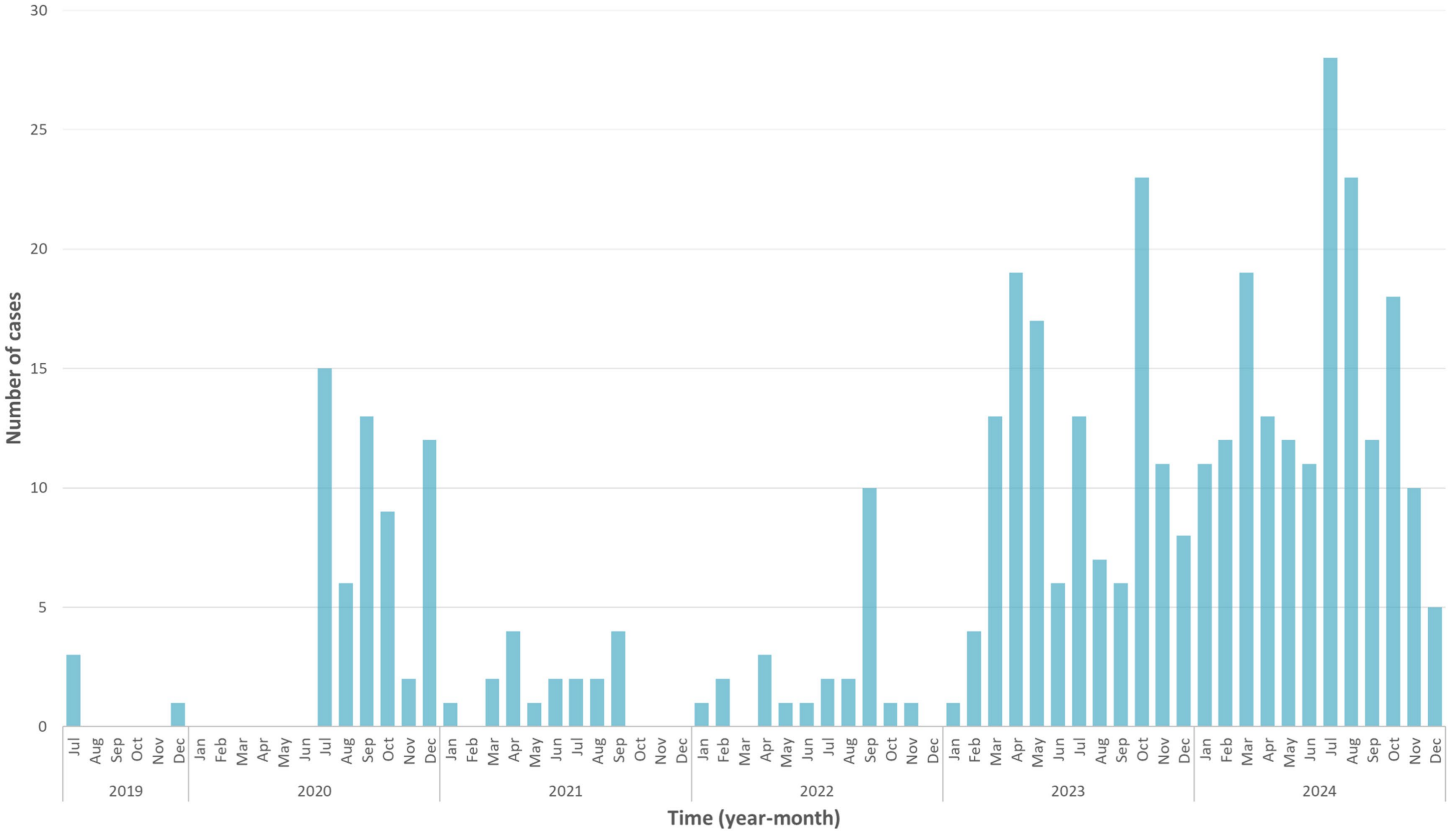
Number of *Candidozyma auris* cases reported to Centre for Health Protection of the Department of Health, 2019–2024.

*C. auris* was first reported in Hong Kong in June 2019, with an upsurge occurring since mid-2020 during the COVID-19 pandemic (Figure 1) (14). From 2019 to September 2023, 41 hospital clusters occurred in Hong Kong, affecting a total of 400 persons, while 8 clusters occurred in RCHEs affecting a total of 67 persons (15). For residents of the RCHE who are diagnosed with *C. auris* at public hospitals, the hospital infection control team would inform the Infection Control Branch (ICB) of the Centre for Health Protection to prepare the RCHE to take care of the residents. ICB staff would provide training and onsite advice on infection control, including placement in a single room or cohorting in the same room or a partitioned area, contact precautions, the use of dedicated medical equipment, and proper cleaning and disinfection of the environment using diluted household bleach (16). Colonization status would be monitored by the Community Geriatric Assessment Teams (CGAT) or Community Nursing Service (CNS) under the Hospital Authority until residents were cleared.

Several studies have shown the fungistatic effect of manuka honey against *Candida* species (17, 18). For example, manuka body wash and mouthwash have been shown to have fungicidal effects against *C. auris* at concentrations to 0.39% (w/v) and 6.25% (v/v) of products supplied for use, respectively (19). Manuka honey belongs to the ‘non-peroxide-based’ honey group, and its main antimicrobial action is contributed by methylglyoxal, which is converted non-enzymatically from the dihydroxyacetone present in the nectar of the flowers of *Leptospermum scoparium* (20). Probiotics and synbiotics offer multifaceted antifungal activities, including biosurfactant and bacteriocin release, organic acid production, nutrient sequestration, hydrogen peroxide secretion, and quorum-sensing interference (21). Evidence from preclinical and clinical studies, including those using *Lactobacillus* and *Saccharomyces,* suggests that probiotics can mitigate *Candida* colonization in the gastrointestinal tract, oral cavity, and vagina, while topical applications remain underexplored (22–28). To shorten the clearance time and reduce transmission risk within the RCHE, we provided a 3-month decolonization therapy using manuka honey body wash and oral probiotics containing *Lactobacillus rhamnosus GG* and *Saccharomyces boulardii* since October 2023 for all RCHE residents diagnosed as *C. auris* carriers in hospitals and discharged to RCHE. In this evaluation, we reported the natural clearance duration of *C. auris* and assessed whether decolonization using manuka honey and probiotics could reduce colonization time among RCHE residents.

## Materials and methods

This retrospective study reviewed *C. auris* colonization cases reported to the Centre for Health Protection, Department of Health, Hong Kong Special Administrative Region, from 1 June 2019 to 31 December 2024. We captured all cases reported by public hospitals. Information on the RCHE, including type (private and non-private), geographic location (Hong Kong, Kowloon and New Territories), capacity (small: <90 beds; medium: 90–160 beds; large: >160 beds) and occupancy, was obtained from the Social Welfare Department. Residents who underwent decolonization were considered the intervention group, while those who did not were considered the control group. Since 1 October 2023, all RCHE residents diagnosed as *C. auris* carriers in hospitals and discharged to RCHE have been provided with decolonization products. They were given to all RCHE residents colonized with *C. auris* without any infection, with a view to shortening the colonization period. The manuka honey body wash contains manuka honey 525 + methylglyoxal, manuka oil 25+, and tea tree oil, applied to residents 2 to 3 times per week. Residents were also given *Lactobacillus Rhamnosus GG* capsules (80 mg), 1 capsule per day, and *Saccharomyces Boulardii* powder (250 mg), 1 sachet per day. RCHE staff were educated on the usage of the decolonization products. Compliance with decolonization was monitored by the CGAT, which visited the RCHE weekly for clinical consultation for medical problems of the RCHE residents. The CGAT would remind the RCHE staff to apply the decolonization products and address their concern if they encountered any problems.

**Figure 2:**
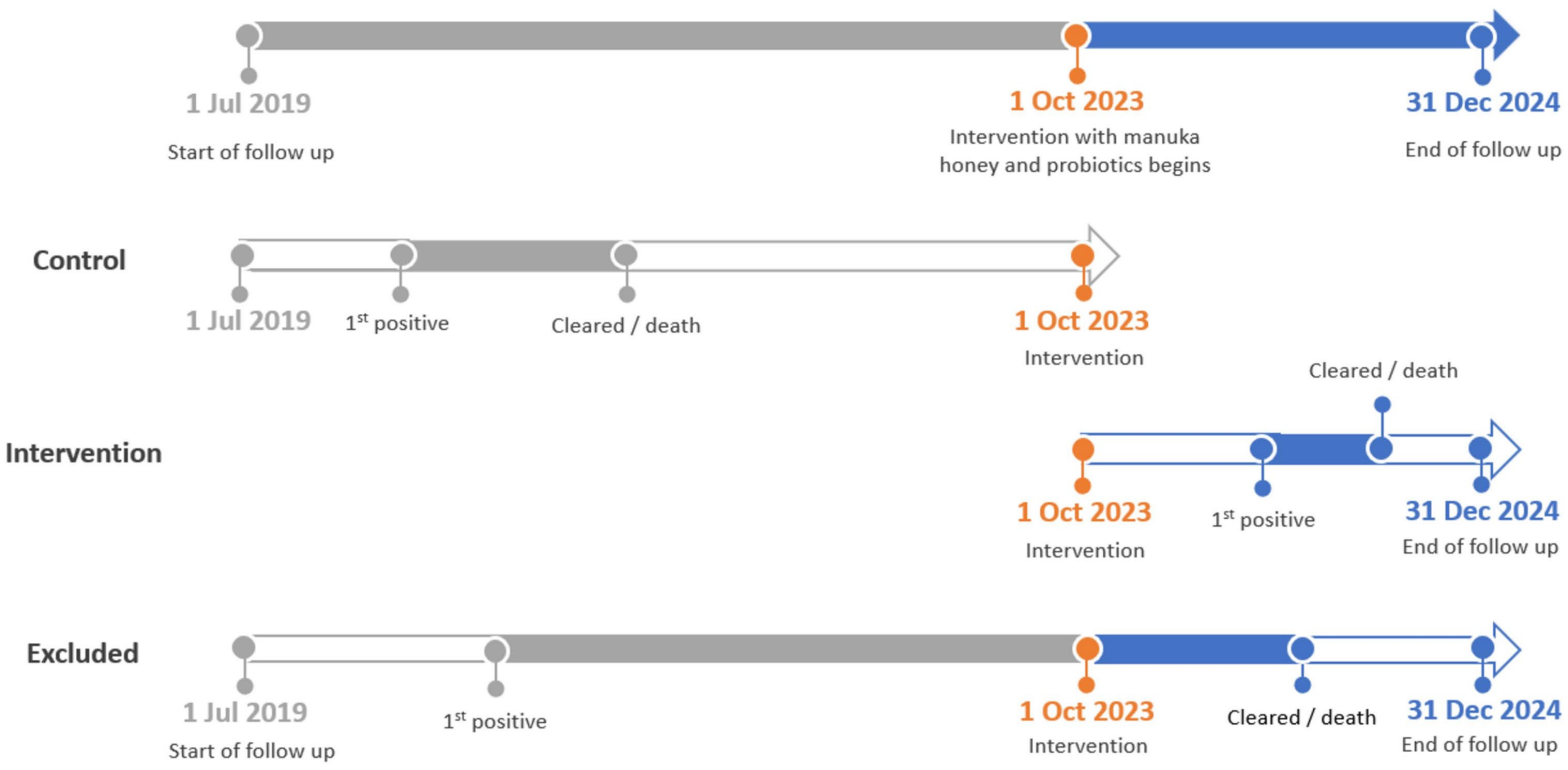
Schematic diagram for follow up of residents of RCHE on the colonisation status of *Candidozyma auris.*

We followed up on the colonization status and outcomes until 31 December 2024, when residents were cleared of *C. auris* or died (Figure 2). Specimens were collected monthly in the RCHE by the outreach team or CGAT/CNS under the Hospital Authority, and sent to hospitals for laboratory testing. Pooled swabs of the resident’s nasal, axilla and groin were taken for *C. auris* culture, and grown at 40–42 °C on CHROMagar™. Diagnostic devices based on matrix-assisted laser desorption or ionization time-of-flight (MALDI-TOF) were used to differentiate *C. auris* from other Candida species. Real-time polymerase chain reaction (PCR) methods were used to facilitate rapid detection of *C. auris* from clinical specimens. To avoid potential false-negative results, the residents should not have been on antifungal medication active against *C. auris* for the past 1 week and topical antiseptics, such as chlorhexidine, for the past 48 h. Residents were considered cleared from *C. auris* if they had three sets of negative specimens.

We compared the demographics and outcomes of the cases and controls using the chi-square test or Fisher’s exact test for categorical data and a t-test for numeric data. Residents were censored at the time of death. Time to *C. auris* clearance was analyzed using time-to-event analysis, with follow-up until 31 December 2024. Residents were excluded from the analysis if the follow-up time consisted of a mixed period of decolonization and non-decolonization since the effect of decolonization could not be clearly determined (Figure 2). The time to *C. auris* clearance was calculated from the collection date of the first positive specimen to the collection date of the third negative specimen. We conducted time-to-event analysis using the Kaplan-Meier methods based on our previous studies for estimation of clearance time for other multidrug-resistant organisms among this group of residents (29). For each time interval, the clearance probability is calculated as the number of subjects colonizing *C. auris* divided by the number of residents at risk. Subjects who have died, dropped out, or cleared are not counted as “at risk,” i.e., subjects who are lost are considered “censored” and are not counted in the denominator. The probability of clearance over time between two groups was compared using the log-rank test. The statistical analysis was performed using R 4.5.0 with a type I error rate of 0.05. As a public health and quality improvement initiative, this surveillance program was exempt from human participant research oversight by the Centre for Health Protection, Department of Health of the HKSAR. The study was performed in accordance with the ethical standards of the Helsinki Declaration, accepted by the World Health Community in 1975 (revised in 2013).

## Results

The monthly number of *C. auris* cases reported is shown in Figure 1, with an upsurge in 2023 and 2024 after it was first identified in 2019. No obvious seasonal trends were observed. A total of 405 cases were included in the analysis, while 139 cases were excluded as their follow-up period included both decolonization and non-decolonization, as mentioned in the method section. The baseline characteristics of those included and excluded from the analysis were similar and showed no statistically significant differences (Table 1). Table 2 summarizes the characteristics of RCHE residents in the intervention group (*n* = 215) and the control group (*n* = 190). The mean age of the intervention and control groups was 80.3 and 80.9 years, respectively (*p* = 0.60). There was no significant difference in sex distribution between the intervention and control groups (*p* = 0.23). A statistically significant difference in case distribution was found between the intervention and control groups (*p* = 0.05). The intervention group had a higher proportion of cases in the New Territories (7.0%) than the control group (2.1%). No statistically significant difference was found in the types of homes between the two groups. Private RCHEs accounted for over 70% (intervention group: 76.3%; control: 69.5%) while non-private RCHEs accounted for 26.9% (intervention group: 23.7%; control: 30.5%). The distribution of RCHE capacity across the three categories (<90, 90–160, and >160) differed significantly between the intervention and control groups (*p* < 0.01), with the intervention group having a higher proportion of cases in small RCHEs (<90 capacity, 48.8%) than the control group (22.6%).

**Table 1:** Baseline characteristics of *Candidozyma auris* cases included and excluded in the analysis.

Characteristics		Included	Excluded	*p*-value
Sex (count (%))	Female	139 (34.3%)	56 (40.3%)	0.24
	Male	266 (65.7%)	83 (59.7%)	
Age (mean (95% CI))		80.6 (79.5–81.8)	79.7 (77.6–81.9)	0.47
RCHE type (count (%))	Private	296 (73.1%)	110 (79.1%)	0.19
	Non-private	109 (26.9%)	29 (20.9%)	

**Table 2:** Characteristics of *Candidozyma auris* cases among intervention and control group, 2019–2024.

Characteristics		Intervention group (*n* = 215)	Control group (*n* = 190)	*p*-value
Age group	<65	20	20	0.63
	65–84	103	82	
	≥85	92	88	
Sex	Female	80	59	0.23
	Male	135	131	
Region	Hong Kong	2	1	0.05
	Kowloon	198	185	
	New Territories	15	4	
RCHE type	Private	164	132	0.15
	Non-private	51	58	
RCHE capacity	<90	105	43	<0.01
	90–160	52	71	
	>160	58	76	
RCHE occupancy	<85%	79	63	0.72
	85–95%	63	61	
	>95%	73	66	

RCHE stands for RCHE.

**Figure 3:**
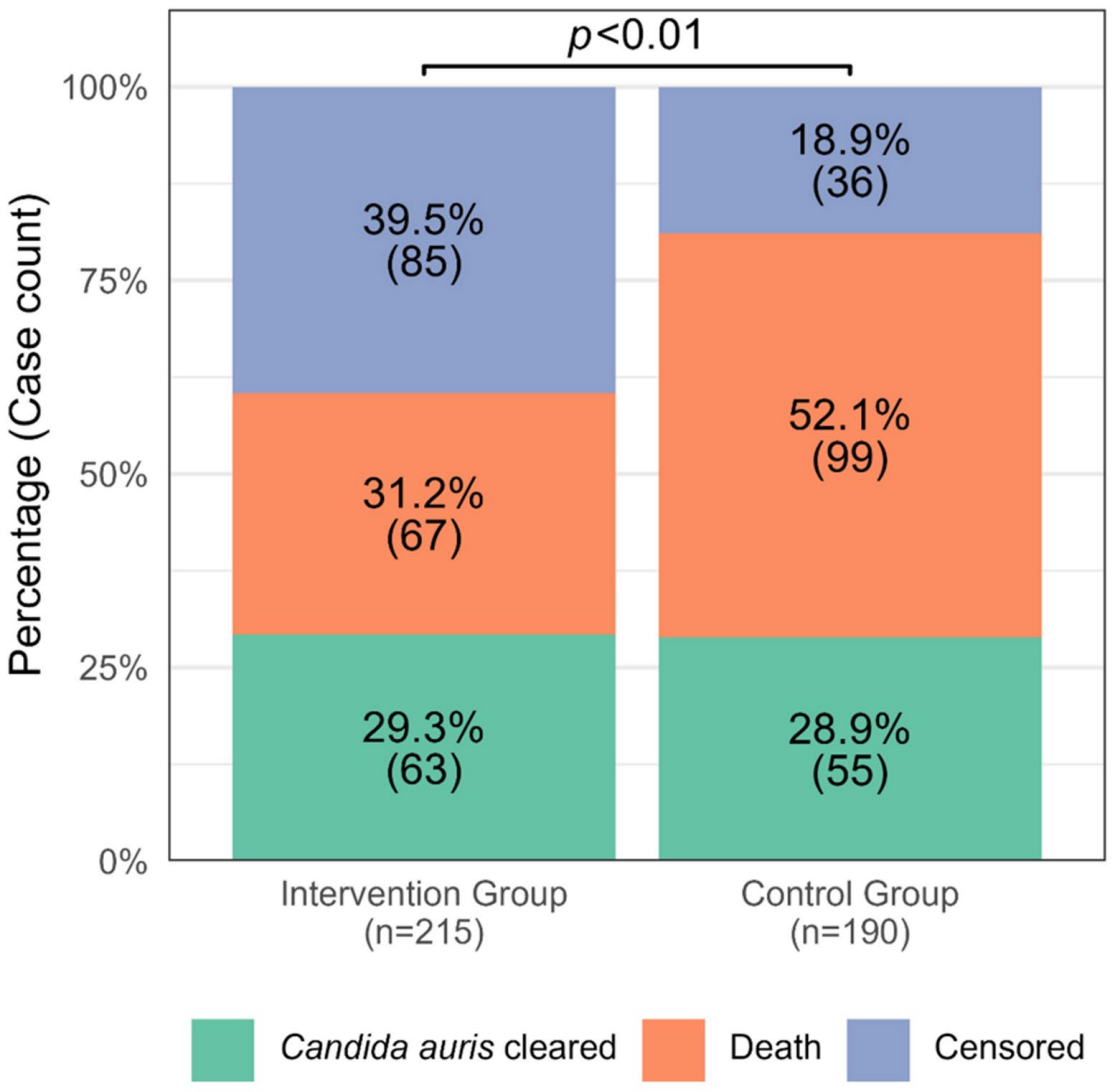
Outcome of *Candidozyma auris* cases with or without decolonization.

**Figure 4:**
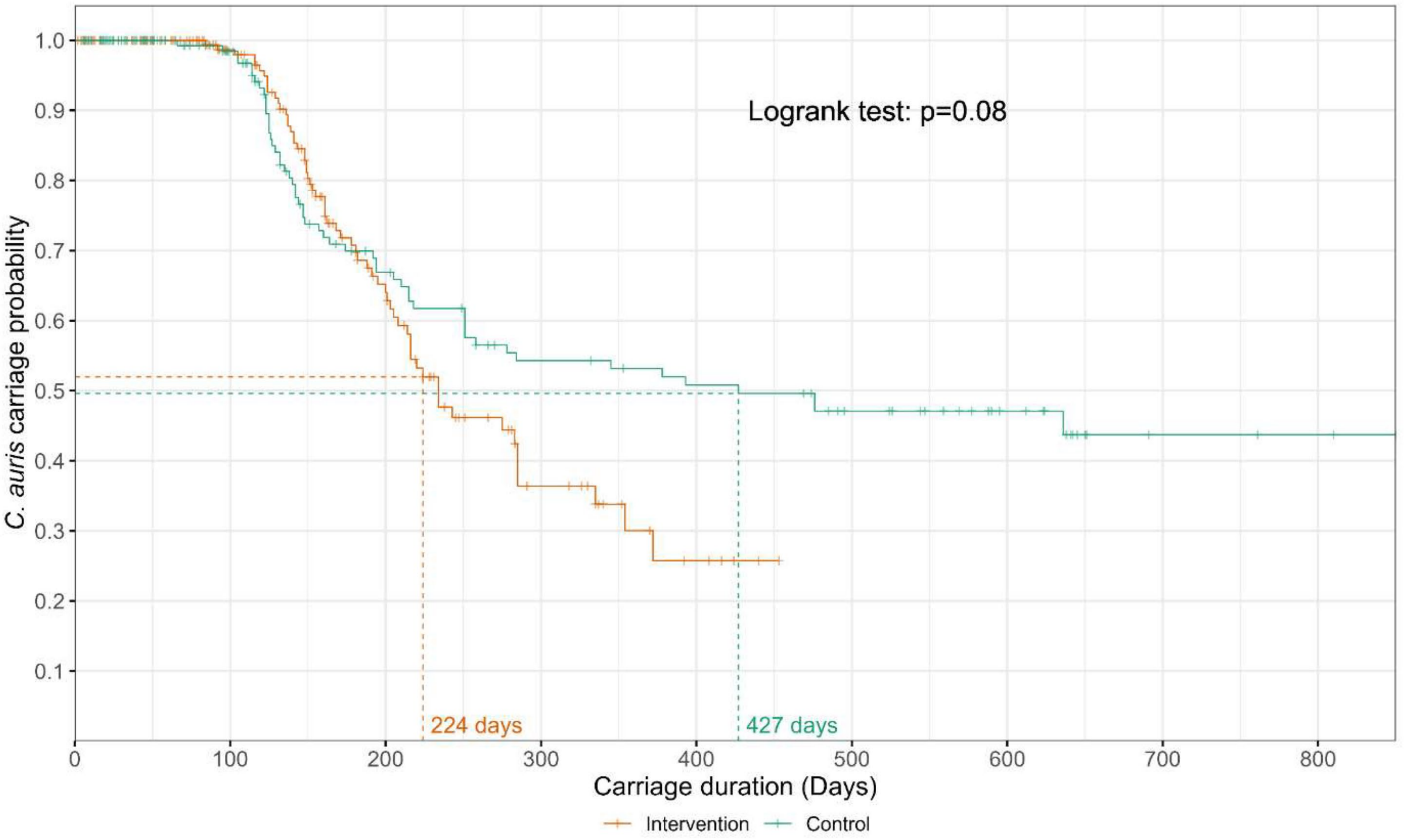
Kaplan-Meier estimate of the duration of positive for *Candidozyma auris* with or without decolonisation.

The number of cases with clearance of *C. auris*, deaths, and censored cases is shown in Figure 3. Notably, the all-cause mortality rate was significantly lower in the intervention group than in the control group (31.2% vs. 52.1%, *p* < 0.01). Figure 4 shows the Kaplan-Meier curve for clearance time for the two groups. The Kaplan-Meier analysis estimated the median time until 50% of RCHE residents cleared *C. auris* colonization to be 224 days in the intervention group and 427 days in the control group. No statistically significant difference in probability of clearance was found between those receiving decolonization and those without (*p* = 0.08).

## Discussion

We estimated the median duration of clearance of *C. auris* among carriers in the RCHE in Hong Kong to be 427 days (more than 13 months), as determined by Kaplan-Meier analysis. The colonization time will determine how long infection control measures should be implemented in these settings. Lengthy duration poses pressure on the heavy workload among RCHE staff in Hong Kong, especially those with tight manpower. In this study, there was no change in clearance time among residents with or without intervention using manuka honey and probiotics over 3 months. Therefore, the prevention of *C. auris* transmission in RCHE mainly depends on the implementation of strict infection control by barrier precautions.

There was scanty literature documenting the clearance time for *C. auris* (30, 31). Our study showed a longer duration of clearance than the results found in a community study with 28 patients, which reported a median time of clearance, defined as having had 2 consecutively negative results, of 8.6 months (interquartile range, 5.7–10.8 months) (31). We believe the present findings are closer to reality as our study included a much larger sample and a long duration of follow-up. The carriage time of *C. auris* is longer than that of other resistant bacteria, such as vancomycin-resistant *Enterococcus*, which was found to be 25 weeks (about 6 months) in a meta-analysis (32). Thermotolerance and osmotolerance are characteristics that may contribute to the persistence and survival of *C. auris* on biotic and abiotic surfaces for long periods of time (3). Besides, *C. auris* biofilms may contribute to the prolonged survival of *C. auris* in the host and environment.

Our analysis of the Kaplan-Meier curve shows a similar rate of clearance in the two groups, particularly during the initial 7 months of follow-up. One would expect there should be a difference in clearance probability within this initial period if decolonization works. There is limited evidence for decolonization of *C. auris* in clinical settings. In an *in vivo* study, manuka honey inhibited *C. auris* at 25% (v/v), and the growth inhibition was dose-dependent (19). The author of the same study reported that in a clinical trial on *C. auris* decolonization using manuka body wash, manuka mouthwash, tea tree oil, povidone-iodine, and nystatin oral suspension in 41 patients, we achieved approximately a 60% success rate, and no adverse effects were reported (19). Manuka honey belongs to the ‘non-peroxide-based’ honey group, and its main antimicrobial action is contributed by methylglyoxal. On the other hand, probiotics are defined as live microorganisms that, when administered in adequate amounts, confer a health benefit on the host (33). The mechanisms by which probiotics protect against infection include producing antimicrobial compounds, for example bacteriocins, and thereby inducing antagonistic effects, aggregation with pathogens, stimulating immunomodulatory cells, improving barrier function of intestinal mucosa, modulating inflammatory responses, competing with the pathogen for binding sites and receptor sites, nutrients, and growth factors (34–36). As probiotics are regarded as generally safe, future research to study their effectiveness in decolonization is encouraged. Should manuka honey baths or oral probiotics be effective in shortening clearance time, there may be a couple of reasons for the non-significant results in the present evaluation. First, this is a retrospective review, and cases and controls were not randomized. Potential confounding factors may account for the non-significant results observed. Second, it is unknown whether the residents or RCHE staff strictly adhered to decolonization. Although the cases and controls originate from different calendar periods, this is unlikely to affect the clearance time of *C. auris,* which should mainly depend on intrinsic factors such as host immunity or colonization pressure, rather than extrinsic factors of the environment or hygiene practices of staff.

Apart from manuka honey and probiotics, chlorhexidine is another product frequently used for decolonization against *C. auris*. Nevertheless, the efficacy against *C. auris* is limited in clinical settings, resulting in persistent skin colonization (37, 38). Furthermore, chlorhexidine is occasionally associated with severe allergic and anaphylactic reactions (39). In addition, octenidine is another synthetic antimicrobial molecule that has been successfully deployed for skin, wound, and mucous membrane antisepsis (40–42). *In vitro* studies have shown that octenidine-based antiseptics significantly reduced *C. auris* viability on intact and wounded human skin, and also demonstrated nearly complete eradication across tested strains (43). These findings provide initial evidence on octenidine-based products in reducing *C. auris* colonization, but future human trials are worthwhile to demonstrate clinical applications.

It was noted that all-cause mortality in the intervention group was significantly lower than in the control group (Figure 3). Since these cases did not have clinical infections due to *C. auris*, it was unlikely due to any potential beneficial effects of decolonization in reducing mortality. The difference in mortality may reflect differences in the follow-up time of the *C. auris* carriers among the intervention and control groups. In a local study of 312 RCHE residents, 107 (34%) died within 1 year of follow-up (44). Another local modeling study projected that 51% of RCHE residents would die in 5 years of follow-up (45). Therefore, it is expected that a longer duration of follow-up might report a higher mortality rate due to common causes of death in the older adults, such as heart diseases or cancers. In the present study, most cases in the intervention group were followed for at least a year, and the mortality rate (31.2%) was comparable to that of a previous local study. In the control group, most residents were followed up for 2 to 4 years, which explains a higher reported mortality of 52.1%.

The current evaluation is well-represented because we included all cases reported by all public hospitals in Hong Kong, so selection bias should be minimal. The long duration of follow-up (longest 60 months) has ensured we detect extreme case of carriage. There was no major problem of compliance observed in the application of decolonization products provided. This evaluation is limited by the retrospective nature and lack of information such as co-morbidity of residents and concurrent medication used for additional analysis. Nevertheless, since the residents were just asymptomatic carriers of *C. auris* rather than having clinical infections, the effect of potential confounders such as anti-fungal agents should be minimal. Besides, follow-up samples were taken at monthly intervals. If more frequent tests had been performed, a more precise clearance time would have been detected. Finally, while it is acknowledged that the use of historical controls might possibly introduce a period effect potentially influenced by enhanced infection control practices in the post-COVID-19 era, time-to-event analysis was utilized to account for varying follow-up durations and censoring. Alternative methods, such as comparing median durations, would exclude censored cases and introduce significant survivor bias and were therefore not applied to the current analysis.

## Conclusion

The long natural clearance time of *C. auris* poses pressure on infection control practices in settings such as RCHE. Our analysis found that decolonization using manuka honey bath and oral probiotics showed no difference in *C. auris* clearance, and therefore did not support routine provision of these products for *C. auris* carriers in RCHE. Further research, such as a randomized controlled trial for manuka honey bath and other treatment modalities, is encouraged to explore the shortening of carriage and transmission risk of this emerging pathogen.

## Data Availability

The raw data supporting the conclusions of this article will be made available by the authors, without undue reservation.

## References

[1] Lockhart SR Etienne KA Vallabhaneni S Farooqi J Chowdhary A Govender NP . Simultaneous emergence of multidrug-resistant Candida auris on 3 continents confirmed by whole-genome sequencing and epidemiological analyses. Clin Infect Dis. (2017) 64:134–40. doi: 10.1093/cid/ciw691, 27988485 PMC5215215

[2] Spivak ES Hanson KE. Candida auris: an Emerging Fungal Pathogen. J Clin Microbiol. (2018) 56:e01588–17. doi: 10.1128/JCM.01588-17, 29167291 PMC5786713

[3] Du H Bing J Hu T Ennis CL Nobile CJ Huang G. Candida auris: epidemiology, biology, antifungal resistance, and virulence. PLoS Pathog. (2020) 16:e1008921. doi: 10.1371/journal.ppat.1008921, 33091071 PMC7581363

[4] Pristov KE Ghannoum MA. Resistance of Candida to azoles and echinocandins worldwide. Clin Microbiol Infect. (2019) 25:792–8. doi: 10.1016/j.cmi.2019.03.028, 30965100

[5] Hata DJ Humphries R Lockhart SR. College of American Pathologists Microbiology Committee. Candida auris: an emerging yeast pathogen posing distinct challenges for laboratory diagnostics, treatment, and infection prevention. Arch Pathol Lab Med. (2020) 144:107–14. doi: 10.5858/arpa.2018-0508-RA, 31169997

[6] World Health Organisation. WHO fungal priority pathogens list to guide research, development and public health action. (2022) Available online at: https://iris.who.int/bitstream/handle/10665/363682/9789240060241-eng.pdf?sequence=1 (Accessed September 2, 2025).

[7] Hübner N Dittmann K Begunk R Kramer A. Infection control measures and prevalence of multidrug-resistant organisms in nonhospital care settings in northeastern Germany: results from a one-day point prevalence study. J Hosp Infect. (2017) 97:234–40. doi: 10.1016/j.jhin.2017.08.002, 28797758

[8] Eveillard M LaFargue S Guet L Mangeol A Piquet J Quenon J-L . Association between institutionalisation and carriage of multiresistant Bacteria in the elderly at the time of admission to a general hospital. Eur J Clin Microbiol Infect Dis. (1999) 18:133–6. doi: 10.1007/s100960050241, 10219578

[9] Ma ES Wong SC Cheng VC Hsu E Chen H Tsui EL. Rising threats of MRSA and Carbapenem-resistant Acinetobacter in residential care homes for the elderly during COVID-19 in Hong Kong. Microorganisms. (2025) 13:1912. doi: 10.3390/microorganisms13081912, 40871416 PMC12388084

[10] Shing S Chen H. Ma ES-k, managing Candida auris outbreak in residential care homes for the elderly (RCHE): key takeaways from an outbreak in Hong Kong. Infect Prev Pract. (2026) 8:100518. doi: 10.1016/j.infpip.2026.100518, 41853692 PMC12992098

[11] Ruiz-Gaitan A Moret AM Tasias-Pitarch M Aleixandre-Lopez AI Martinez-Morel H Calabuig E . An outbreak due to Candida auris with prolonged colonisation and candidaemia in a tertiary care European hospital. Mycoses. (2018) 61:498–505. doi: 10.1111/myc.12781, 29655180

[12] Huang X Welsh RM Deming C Proctor DM Thomas PJ Comparative Sequencing Program NISC . Skin metagenomic sequence analysis of early Candida auris outbreaks in U.S. Nurs. Homes mSphere. (2021) 6:e0028721. doi: 10.1128/mSphere.00287-21, 34346704 PMC8386442

[13] Ruiz-Gaitan A Martinez H Moret AM Calabuig E Tasias M Alastruey-Izquierdo A . Detection and treatment of Candida auris in an outbreak situation: risk factors for developing colonization and candidemia by this new species in critically ill patients. Expert Rev Anti-Infect Ther. (2019) 17:295–305. doi: 10.1080/14787210.2019.1592675, 30922129

[14] Ma ESK Kung KH Chen H. Combating antimicrobial resistance during the COVID-19 pandemic. Hong Kong Med J. (2021) 27:396–8. doi: 10.12809/hkmj215124, 34785620

[15] Centre for Health Protection, Department of Health, Hong Kong Special Administrative Region. Review of Candida auris in Hong Kong. Commun Dis Watch (2023). 19:38–39. Available online at: https://www.chp.gov.hk/files/pdf/cdw_v19_7.pdf (Accessed on September 2, 2025).

[16] Centre for Health Protection, Department of Health, Hong Kong Special Administrative Region. Infection control advice on Multi-Drug Resistant Organisms (MDROs) for Residential Care Homes for the Elderly (RCHEs). Available online at: https://www.chp.gov.hk/files/pdf/infection_control_advice_on_mdro_for_rche_eng.pdf (Accessed September 2, 2025).

[17] Anand S Deighton M Livanos G Pang ECK Mantri N. Agastache honey has superior antifungal activity in comparison with important commercial honeys. Sci Rep. (2019) 9:18197. doi: 10.1038/s41598-019-54679-w, 31796803 PMC6890684

[18] Fernandes L Oliveira A Henriques M Rodrigues ME. Honey as a strategy to fight *Candida tropicalis* in mixed-biofilms with *Pseudomonas aeruginosa*. Antibiotics (Basel). (2020) 9:43. doi: 10.3390/antibiotics9020043, 31973242 PMC7168267

[19] Wu WG Luk KS Hung MF Tsang WY Lee KP Lam BH . Antifungal efficacy of natural antiseptic products against Candida auris. Med Mycol. (2024) 62:myae060. doi: 10.1093/mmy/myae060, 38936838

[20] Mandal MD. Mandal S honey: its medicinal property and antibacterial activity. Asian Pacific J Trop Biomed. (2011) 1:154–60. doi: 10.1016/S2221-1691(11)60016-6, 23569748 PMC3609166

[21] Fayed B. Harnessing probiotics to combat acquired resistance in Candida auris: emerging mechanisms and formulation strategies. Probiotics Antimicrob Proteins. (2026). doi: 10.1007/s12602-025-10913-8, 41806262

[22] Wu Y Hu S Wu C Gu F Yang Y. Probiotics: potential novel therapeutics against fungal infections. Front Cell Infect Microbiol. (2021) 11:793419. doi: 10.3389/fcimb.2021.793419, 35127557 PMC8813855

[23] Kunyeit L Kurrey NK Anu-Appaiah KA Rao RP. Probiotic yeasts inhibit virulence of non-albicans Candida species. MBio. (2019) 10:e02307–19. doi: 10.1128/mBio.02307-19, 31615960 PMC6794482

[24] Gerbaldo GA Barberis C Pascual L Dalcero A Barberis L. Antifungal activity of two Lactobacillus strains with potential probiotic properties. FEMS Microbiol Lett. (2012) 332:27–33. doi: 10.1111/j.1574-6968.2012.02570.x, 22497448

[25] Adnan M Siddiqui AJ Noumi E Ashraf SA Awadelkareem AM Hadi S . Biosurfactant Derived from probiotic *Lactobacillus acidophilus* exhibits broad-spectrum antibiofilm activity and inhibits the quorum sensing-regulated virulence. Biomol Biomed. (2023) 23:1051–68. doi: 10.17305/bb.2023.9324, 37421468 PMC10655870

[26] Paniágua AL Correia AF Pereira LC de Alencar BM Silva FBA Almeida RM . Inhibitory effects of *Lactobacillus casei* Shirota against both Candida auris and Candida spp. isolates that cause vulvovaginal candidiasis and are resistant to antifungals. BMC Complement Med Ther. (2021) 21:237. doi: 10.1186/s12906-021-03405-z, 34556109 PMC8461885

[27] Ullah A Orij R Brul S Smits Gertien J. Quantitative analysis of the modes of growth inhibition by weak organic acids in *Saccharomyces cerevisiae*. Appl Environ Microbiol. (2012) 78:8377–87. doi: 10.1128/AEM.02126-12, 23001666 PMC3497387

[28] Peetermans A Foulquié-Moreno MR Thevelein JM. Mechanisms underlying lactic acid tolerance and its influence on lactic acid production in *Saccharomyces cerevisiae*. Microb Cell. (2021) 8:111–30. doi: 10.15698/mic2021.06.751, 34055965 PMC8144909

[29] Ma ES Lai RW Chuang VW Shing BS Lui L Hsu E . Natural clearance of colonization with vancomycin-resistant Enterococcus and carbapenemase-producing Enterobacterales: a 13-year study among territory-wide residents of residential care home for the elderly in Hong Kong. Antimicrob Resist Infect Control. (2026) 15:31. doi: 10.1186/s13756-026-01702-1, 41618378 PMC12930830

[30] Yadav A Singh A Wang Y Haren MHV Singh A de Groot T . Colonisation and transmission dynamics of Candida auris among chronic respiratory diseases patients hospitalised in a chest hospital, Delhi, India: A Comparative analysis of whole genome Sequencing and microsatellite typing. J Fungi (Basel). (2021) 7:81. doi: 10.3390/jof7020081, 33530297 PMC7910912

[31] Bergeron G Bloch D Murray K Kratz M Parton H Ackelsberg J . Candida auris colonization after discharge to a community setting: new York City, 2017-2019. Open forum. Infect Dis. (2020) 8:ofaa620. doi: 10.1093/ofid/ofaa620, 33511238 PMC7814391

[32] Shenoy ES Paras ML Noubary F Walensky RP Hooper DC. Natural history of colonization with methicillin-resistant *Staphylococcus aureus* (MRSA) and vancomycin-resistant Enterococcus (VRE): a systematic review. BMC Infect Dis. (2014) 14:177. doi: 10.1186/1471-2334-14-177, 24678646 PMC4230428

[33] Salminen S Collado MC Endo A Hill C Lebeer S Quigley EMM . The international scientific Association of Probiotics and Prebiotics (ISAPP) consensus statement on the definition and scope of postbiotics. Nat Rev Gastroenterol Hepatol. (2021) 18:649–67. doi: 10.1038/s41575-021-00440-6 33948025 PMC8387231

[34] Kosgey JC Jia L Fang Y Yang J Gao L Wang J . Probiotics as antifungal agents: experimental confirmation and future prospects. J Microbiol Methods. (2019) 162:28–37. doi: 10.1016/j.mimet.2019.05.001, 31071354

[35] Amara AA Shibl A. Role of probiotics in health improvement, infection control and disease treatment and management. Saudi Pharm J. (2015) 23:107–14. doi: 10.1016/j.jsps.2013.07.001, 25972729 PMC4421088

[36] Deidda F Amoruso A Allesina S Pane M Graziano T Del Piano M . *In vitro* activity of *Lactobacillus fermentum* LF5 against different Candida species and *Gardnerella vaginalis*: A new perspective to approach mixed vaginal infections? J Clin Gastroenterol. 2015:S168–70. doi: 10.1097/MCG.0000000000000692, 27741167

[37] Schelenz S Hagen F Rhodes JL Abdolrasouli A Chowdhary A Hall A . First hospital outbreak of the globally emerging Candida auris in a European hospital. Antimicrob Resist Infect Control. (2016) 5:35. doi: 10.1186/s13756-016-0132-5, 27777756 PMC5069812

[38] Septimus EJ Schweizer ML. Decolonization in prevention of health care-associated infections. Clin Microbiol Rev. (2016) 29:201–22. doi: 10.1128/CMR.00049-15, 26817630 PMC4786886

[39] Chiewchalermsri C Sompornrattanaphan M Wongsa C Thongngarm T. Chlorhexidine allergy: current challenges and future prospects. J Asthma Allergy. (2020) 13:127–33. doi: 10.2147/JAA.S207980, 32210588 PMC7069565

[40] Dettenkofer M Wilson C Gratwohl A Schmoor C Bertz H Frei R . Skin disinfection with octenidine dihydrochloride for central venous catheter site care: a double-blind, randomized, controlled trial. Clin Microbiol Infect. (2010) 16:600–6. doi: 10.1111/j.1469-0691.2009.02917.x, 19686276

[41] Eisenbeiß W Siemers F Amtsberg G Hinz P Hartmann B Kohlmann T . Prospective, double-blinded, randomised controlled trial assessing the effect of an Octenidine-based hydrogel on bacterial colonisation and epithelialization of skin graft wounds in burn patients. Int J Burns Trauma. (2012) 2:71–9. 23071904 PMC3462524

[42] Vanscheidt W Harding K Téot L Siebert J. Effectiveness and tissue compatibility of a 12-week treatment of chronic venous leg ulcers with an octenidine based antiseptic--a randomized, double-blind controlled study. Int Wound J. (2012) 9:316–23. doi: 10.1111/j.1742-481X.2011.00886.x, 22074592 PMC7951026

[43] Cerbu D Seiser S Phan-Canh T Moser D Freystätter C Matiasek J . Octenidine effectively reduces Candida auris colonisation on human skin. Sci Rep. (2025) 15:27034. doi: 10.1038/s41598-025-11914-x, 40715367 PMC12297581

[44] Luk JK Chan WK Ng WC Chiu PK Ho C Chan TC . Mortality and health services utilisation among older people with advanced cognitive impairment living in residential care homes. Hong Kong Med J. (2013) 19:518–24. doi: 10.12809/hkmj133951, 24096360

[45] Lee JS Chau PP Hui E Chan F Woo J. Survival prediction in nursing home residents using the minimum data set subscales: ADL self-performance hierarchy, cognitive performance and the changes in health, end-stage disease and symptoms and signs scales. Eur J Pub Health. (2009) 19:308–12. doi: 10.1093/eurpub/ckp006, 19221020

